# DCHS1, Lix1L, and the Septin Cytoskeleton: Molecular and Developmental Etiology of Mitral Valve Prolapse

**DOI:** 10.3390/jcdd9020062

**Published:** 2022-02-17

**Authors:** Kelsey S. Moore, Reece Moore, Diana B. Fulmer, Lilong Guo, Cortney Gensemer, Rebecca Stairley, Janiece Glover, Tyler C. Beck, Jordan E. Morningstar, Rachel Biggs, Rupak Muhkerjee, Alexander Awgulewitsch, Russell A. Norris

**Affiliations:** 1 Department of Regenerative Medicine and Cell Biology, Medical University of South Carolina, Charleston, SC 29425, USA; kelsey@histowiz.com (K.S.M.); moorere@musc.edu (R.M.); guol@musc.edu (L.G.); gensemer@musc.edu (C.G.); stairley@musc.edu (R.S.); gloverja@musc.edu (J.G.); beckt@musc.edu (T.C.B.); morningj@musc.edu (J.E.M.); biggsr@musc.edu (R.B.); awgulewa@musc.edu (A.A.); 2 Perelman School of Medicine, University of Pennsylvania, Philadelphia, PA 19104, USA; diana.fulmer@pennmedicine.upenn.edu; 3 Department of Surgery, Medical University of South Carolina, Charleston, SC 29425, USA; mukherr@musc.edu

**Keywords:** septin, DCHS1, cytoskeleton, mitral valve prolapse, heart valve development

## Abstract

Mitral valve prolapse (MVP) is a common cardiac valve disease that often progresses to serious secondary complications requiring surgery. MVP manifests as extracellular matrix disorganization and biomechanically incompetent tissues in the adult setting. However, MVP has recently been shown to have a developmental basis, as multiple causal genes expressed during embryonic development have been identified. Disease phenotypes have been observed in mouse models with human MVP mutations as early as birth. This study focuses on the developmental function of DCHS1, one of the first genes to be shown as causal in multiple families with non-syndromic MVP. By using various biochemical techniques as well as mouse and cell culture models, we demonstrate a unique link between DCHS1-based cell adhesions and the septin-actin cytoskeleton through interactions with cytoplasmic protein Lix1-Like (LIX1L). This DCHS1-LIX1L-SEPT9 axis interacts with and promotes filamentous actin organization to direct cell-ECM alignment and valve tissue shape.

## 1. Introduction

Mitral Valve Prolapse (MVP) is one of the most common forms of cardiac valve disease, affecting ~2–3% of the human population. Nearly 1 in 10 MVP patients will require surgery for valve repair or replacement, and it is currently the fastest growing cardiovascular intervention in the Western world [1,2]. MVP is characterized by disrupted extracellular matrix (ECM) resulting in biomechanically incompetent mitral valve leaflets that are no longer able to close during ventricular systole. Progression of MVP can lead to serious secondary complications such as arrythmias, heart failure and sudden cardiac death. Nonsurgical treatments for MVP do not exist and therapeutic efforts have been hindered by an incomplete understanding of its fundamental causes.

This study focuses on a previously identified MVP causal gene, DCHS1 [3], with an aim to establish intracellular pathways mediated by this large, atypical cadherin protein. Two heterozygous protein altering DCHS1 variants were identified in families of non-syndromic MVP and include single nucleotide variants (SNVs) p.R2513H and p.R2330C in the extracellular domain of DCHS1. It was demonstrated that these loss-of-function DCHS1 mutations cause haploinsufficiency and an MVP phenotype in both human and mouse models [3]. Additionally, a recent genetic study revealed that up to 24% of MVP patients have disease-contributing variants in DCHS1 [4]. These data suggest that DCHS1 variants significantly affect cardiac valve pathology and support a broad role for DCHS1 in the etiology of MVP in families as well as in the MVP population.

DCHS1 is the human homologue of the *Drosophila* cell polarity gene, *dachsous* (*ds*), and is translated as a 365 kDa atypical cadherin protein [3,5]. Although previous reports have established a role for DCHS1 in valve morphogenesis in mice and patients with MVP [3], intracellular mechanisms resulting from altered DCHS1-based cell–cell adhesions remain unknown. Previous reports have implicated DCHS1 in various developmental diseases including neurological disorders such as bipolar disorder [6], neuronal heterotopia [7,8] and hypothalamic-pituitary development [9]. Additional studies have established DCHS1 in kidney development [10] and bone development affecting Van Maldergem syndrome [11] and sternum morphogenesis [10,12]. These defects were shown to be a result of DCHS1 heterotypic interactions with atypical cadherin, FAT4 (FAT Atypical Cadherin 4) affecting planar cell polarity (PCP) members. Bosveld et al. also showed that Dachsous and Fat polarize myosin, *Dachs*, in Drosophila as observed previously [13,14] and confer significance in tissue morphogenesis [15]. While it is possible that PCP mechanisms are involved in mitral valve development, little is known regarding the presence of these pathways in the valve. With a combination of in vivo and in vitro analyses, molecular interactions consisting of DCHS1 and cytoplasmic proteins Lix1-Like (LIX1L) and septin-9 (SEPT9) were identified. Perturbation of this complex disrupts the actin cytoskeleton and causes severe consequences on cell-tissue organization and valvulogenesis.

## 2. Results

### 2.1. Identification of DCHS1-LIX1L-SEPT9 (DLS) Protein Complex

**Figure 1:**
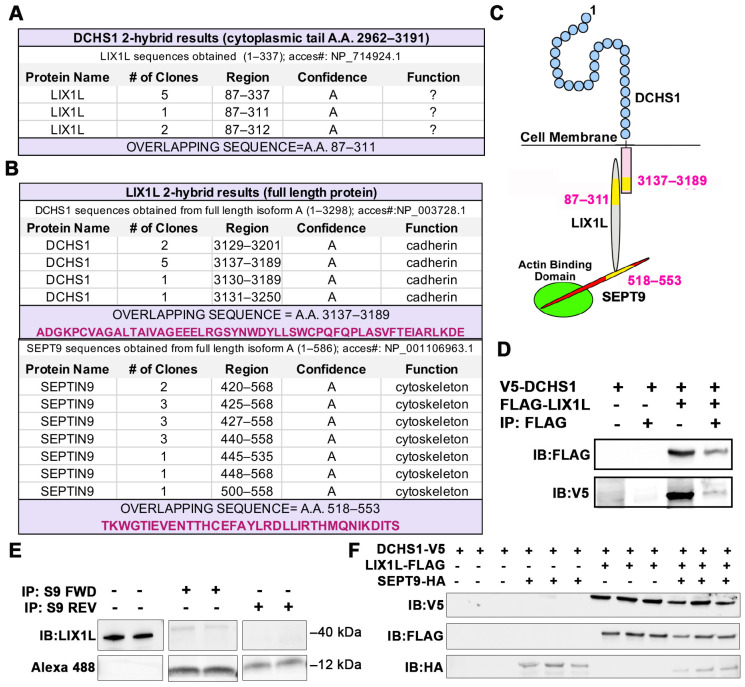
Identification of DCHS1-LIX1L-SEPT9 (DLS) protein complex. (**A**) Yeast two-hybrid (Y2H) screen using cytoplasmic tail (A.A. 2962–3191) of Dachsous Cadherin Related-1 (DCHS1) as bait reveals Lix1-Like (LIX1L). (**B**) Y2H with full-length LIX1-Like (LIX1L) identifies DCHS1 and septin-9 (SEPT9) as binding partners. In A and B, confidence scores represent likelihood of an interaction, with A being the highest level of confidence (See Methods for details). (**C**) Diagram depicting interacting proteins and binding domains. (**D**) Co-Immunoprecipitation (co-IP) and immunoblotting (IB) analysis with DCHS1-V5 and LIX1L-FLAG transfected in HEK293T cells confirms protein interaction. (**E**) Co-IP and IB of cVIC protein lysate treated with 10 μg peptide mimicking the LIX1L-SEPT9 binding domain (S9-FWD:5-FAM-YGRKKRRQRRR-Ahx TKWGTIEVENTTHCEFAYLRDLLIRTHMQNIKDIT-Lys(Biotin), S9-REV:5-FAM-YGRKKRRQRRR-Ahx-TIDKINQMHTRILLDRLYAFECHTTNEVEITGWKT-Lys(Biotin)). (**F**) IB of DCHS1-V5, LIX1L-FLAG and SEPT9-HA co-transfected into HEK293T cells depicts stabilization of DCHS1 protein only in the presence of LIX1L.

To elucidate novel protein interactors of DCHS1 beyond the well-known FAT4 binding partner, a yeast two-hybrid (Y2H) screen was performed using the cytoplasmic portion of DCHS1 as bait. In an initial screen, we used the cytoplasmic tail of DCHS1 containing amino acids 2962–3191 of human DCHS1 (NM_003737.3) against a human placenta library containing 110 million clones. The cytoplasmic protein Lix1-Like (LIX1L) emerged as an abundant binding partner (Figure 1A). Overlap of numerous clones that emerged from the two-hybrid screen resulted in identification of an interaction motif within the 87–311 amino acid stretch of LIX1L. The interaction was confirmed with co-immunoprecipitation assays in HEK293T cells (Figure 1D). A second two-hybrid screen was performed using a shorter piece of the cytoplasmic tail (amino acids 2962–3130) in which no clones of high significance were achieved. This likely placed the LIX1L interaction motif within amino acids 3130–3191 of DCHS1. While this identification established a novel interaction between DCHS1 and LIX1L in the heart, little is known regarding the function of LIX1L as a cytoplasmic protein. Previous studies have implicated its role in bile acid synthesis in cholestatic liver injury [16] and in cancer cell proliferation [17], protein functions that have not been shown to significantly affect mitral morphogenesis. To uncover LIX1L-mediated pathways, an additional two-hybrid screen was performed using full-length LIX1L protein as bait against the same human heart library. DCHS1 was identified as a binding partner and the interaction was refined to A.A. 3137–3189 within the cytoplasmic tail, validating a direct interaction between DCHS1 and LIX1L (Figure 1B). In addition, septin-9 (SEPT9), a member of the septin cytoskeletal family of proteins, emerged as direct binding partner of LIX1L (Figure 1B). Sequence analyses of overlapping clones revealed a short, 36 amino acid binding domain (A.A. 518–553) within the carboxyl-tail of SEPT9 as the LIX1L interaction motif. A peptide was synthesized, conjugated with biotin and 5-carboxyfluorescein (5-FAM) and used in confirmatory co-immunoprecipitation assays in chicken valve interstitial cells (cVICs) (Figure 1E). These data demonstrate that this short peptide in SEPT9 harbors the LIX1L interaction domain.

Expression of DCHS1 in HEK293T cells failed to result in detectable presence of protein when transfected alone or in addition with SEPT9 (Figure 1F). However, when LIX1L was co-expressed with DCHS1, robust expression of DCHS1 and LIX1 was evident. Co-expression of DCHS1 and SEPT9 was insufficient to result in stable expression, demonstrating the necessity of LIX1L in stabilizing the complex as assayed by the presence of each of the proteins. Requirement for full assembly of this complex was further suggested in vivo, in which SEPT9 expression is dependent on the presence of DCHS1 and LIX1L in developing valves (Figure S1). 

**Figure 2:**
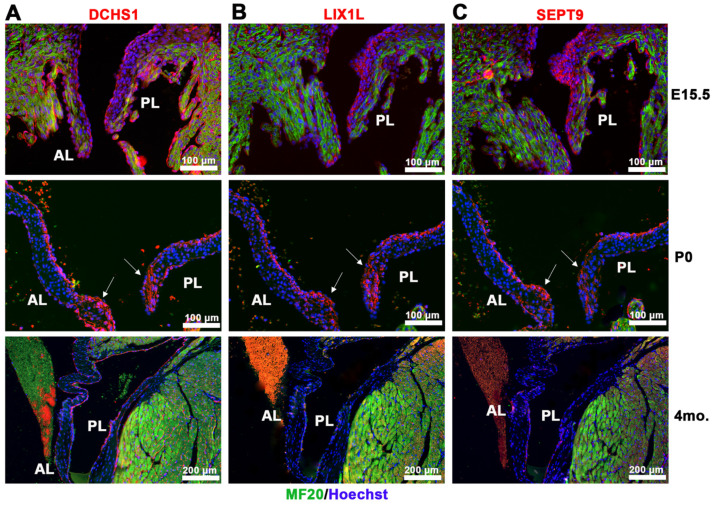
Spatio-temporal expression of DCHS1-LIX1L-SEPT9 proteins throughout development. IHC staining of (**A**) DCHS1, (**B**) LIX1L and (**C**) SEPT9 (red), MF20 (green), and nuclei (Hoechst, blue) on sister sections of E15.5, P0 and four-month-old wildtype mouse valve tissue reveals similar expression throughout the interstitium with localization to the leaflet tips by P0 (arrows) and restriction to the endothelium through adulthood. AL = anterior leaflet, PL = posterior leaflet.

Co-expression of components of the DCHS1-LIX1L-SEPT9 (DLS) complex in vivo revealed similar expression domains and cell types of each protein during valve morphogenesis. Immunohistochemical (IHC) staining of DCHS1, LIX1L and SEPT9 illustrates expression throughout the valve from embryonic day 15.5 (E15.5) to postnatal day zero (P0) with similar localizations to the valve and myocardial endothelium by four months of age (Figure 2). Expression of each protein within the valve at these time points is primarily restricted to endothelial cells and mesenchymal cells within the atrialis and the valve tips, regions known to express high levels of proteoglycans. True co-localization of each of the proteins is currently not possible due to antibody species being the same for each protein. Nonetheless, the expression of each protein between sister sections is remarkably similar.

### 2.2. DCHS1 and Lix1L Genetically Interact during Valve Development

**Figure 3:**
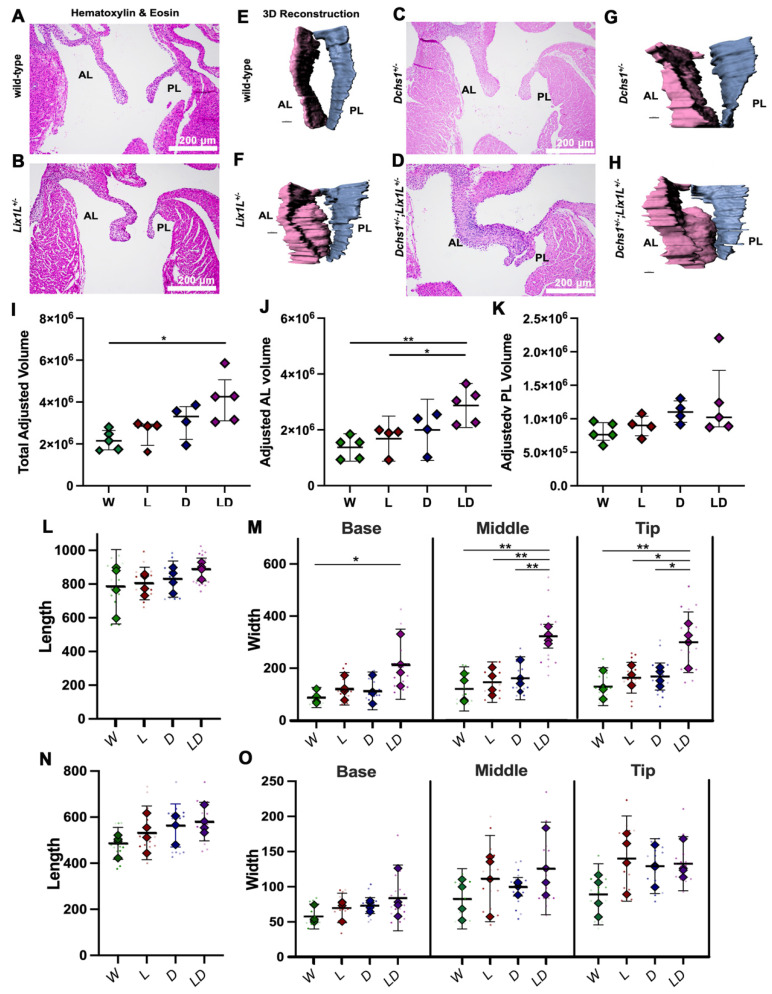
Epistasis analysis reveals DCHS1-LIX1L genetic interaction at postnatal day zero. (**A**–**D**) Hematoxylin and eosin (H&E) staining and (**E**–**H**) 3D reconstructions of postnatal day zero (P0) mitral valves isolated from wildtype (W), single heterozygote (LIX1L^+/−^, L or DCHS1^+/−^, D) and compound heterozygote (LIX1L^+/−^;DCHS1^+/−^, LD) mice reveal thickening of single heterozygote and compound heterozygote anterior leaflets (AL) compared to wildtype control littermates. (**I**–**K**) Total mitral valve volume measurements from 3D-reconstructed leaflet surfaces demonstrate a significant increase in compound heterozygotes AL leaflets compared to wildtype, with no significance observed in posterior leaflet (PL) volumes. Two-dimensional measurements of length (**L**,**N**) and width (**M**,**O**) along the leaflet reveals that changes in leaflet volume are due to significant leaflet thickening in the base and tip of the (**L**,**M**) anterior leaflets of compound heterozygotes versus single heterozygotes or wildtypes. (**N**,**O**) No significant differences were observed in the posterior leaflet of these mice. N = 4–5 animals per genotype, 2D measurements completed on four sections per animal and depicted by dots, averages are plotted as diamonds, * *p* < 0.05, ** *p* < 0.005 with One-Way Anova.

Due to the physical interaction between DCHS1 and LIX1L combined with their similar expression patterns during valvulogenesis, we tested whether a genetic interaction between DCHS1 and LIX1L exists in vivo in mice. If epistasis occurs between DCHS1 and LIX1L, compound heterozygosity is likely to have a synergistic effect on observed phenotypes. Generation of compound heterozygote mice (DCHS1^+/−^; LIX1L^+/−^) revealed significant leaflet enlargement at P0 compared to single heterozygotes or wildtype controls as depicted with Hematoxylin and Eosin (H&E) staining and 3D reconstructions (Figure 3A–H). Quantification of valve volume measurements showed that compound heterozygote leaflets are significantly larger than wildtype control littermates as a result of increased anterior leaflet volume (Figure 3I–K). Two dimensional measurements of valve length and width along the leaflets revealed that enlargement is due to thickening rather than lengthening in DCHS1- and LIX1L-deficient valves. Compound heterozygote anterior leaflets were significantly wider in the mid and tip region compared to single heterozygotes or controls, suggesting a synergistic effect on valve shape and tissue morphogenesis in our mice (Figure 3I). No significant increases were observed in the posterior leaflet at this time point (Figure 3L,N,O). These data suggest that DCHS1 and LIX1L interact within the same pathway to affect valve morphogenesis in vivo. 

**Figure 4:**
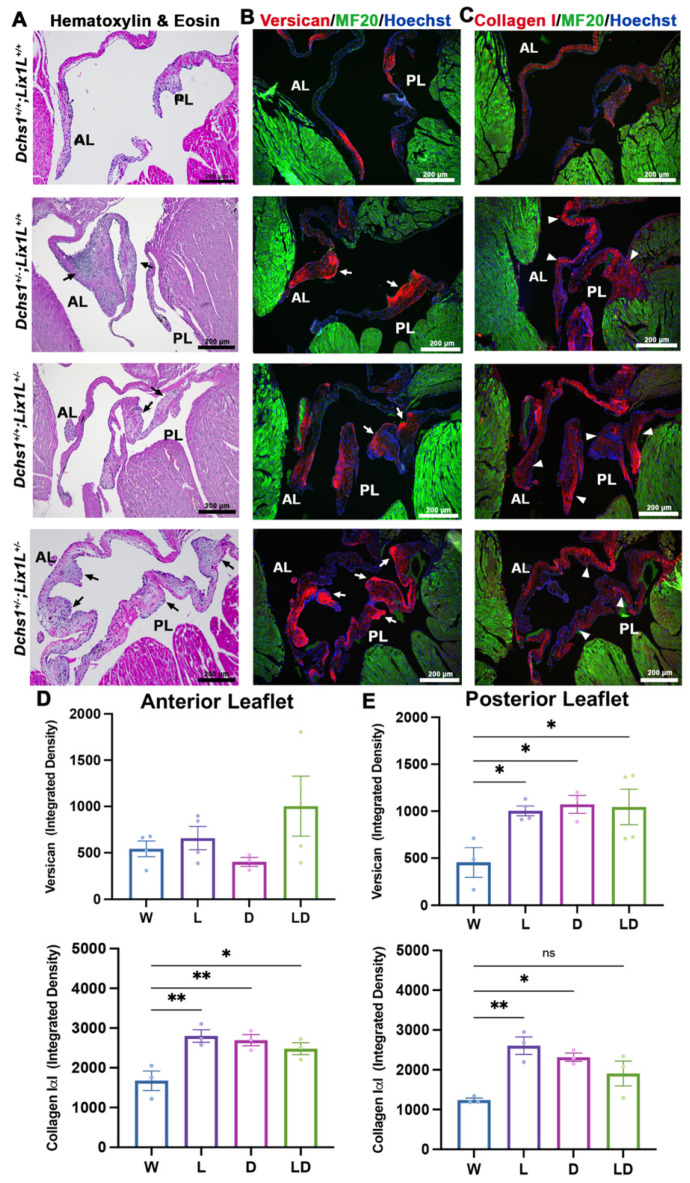
Adult heterozygotes display mitral valve disease phenotype of myxomatous degeneration. (**A**) Representative H&E staining of 11-month-old wildtype (W), single (L, D) and compound heterozygote (LD) mitral valves depict thickening and bulging of single and compound heterozygotes anterior and posterior leaflets (AL, PL) (black arrows). Immunohistochemical (IHC) staining of (**B**) Versican (red) and (**C**) Collagen 1α1 (red), myocardium (MF20, green) and nuclei (Hoechst, blue) reveals increased extracellular matrix deposition and disorganization (white arrows and arrowheads) in single or compound heterozygotes. (**D**,**E**) Quantification of ECM staining intensity reveals statistically significant increases in single or compound heterozygote leaflets compared to wildtype controls, indications of myxomatous degeneration. N = 3 per genotype, depicted with mean and SEM, * *p* < 0.05, ** *p* < 0.005 with One Way Anova.

To test whether altering developmental interactions between DCHS1 and LIX1L was sufficient to engender an adult disease phenotype, echocardiographic and histopathology was performed on adult mice (3, 6 and 11 months). Echocardiography revealed slightly increased prevalence (data not shown) of observed prolapse of either the anterior and posterior leaflets in compound heterozygotes compared to single heterozygotes. Furthermore, measurements of 2D images revealed significant increases in left atrium diameter, a potential surrogate marker or physiological consequence of mitral regurgitation [18,19] (Figure S2). In concordance with observed leaflet thickening in echocardiographic images, histopathological analyses performed on 11-month-old tissue revealed significant enlargements and bulging along both leaflets in single and compound heterozygotes as observed with H&E staining (Figure 4A). Disorganized and increased extracellular matrix deposition, also known as myxomatous degeneration, has been observed in patients and mice with MVP [20] and was assayed with immunohistochemical staining of collagen I and versican. Significant increases in both collagen I and versican were observed in single and compound heterozygotes along the anterior and posterior leaflets (Figure 4) and support previous ECM phenotypes observed in DCHS1^+/−^ mice [3] and in pre-valvular cells derived from MVP patients with p.R2330C SNV [21]. Together, these data demonstrate that DCHS1-LIX1L interactions are critical for ensuring proper regulation of valve tissue structure in vivo.

### 2.3. DCHS1-LIX1L-SEPT9 Is Linked to the Actin Cytoskeleton and Promotes Polymerization

Increasingly recognized as cytoskeletal components, septins are highly conserved GTP-binding, filamentous proteins that have been shown to regulate and interact with actin, microtubules and phospholipids [22]. Septins have been identified in various cellular processes including polarity, mechanotransduction, ciliogenesis and axon growth, among others [23]. As a result, septins have been identified in various diseases, but remain vastly understudied in the context of cardiac development and disease [24]. Figure S3 illustrates the RNA expression of septins 2, 5, 6, 7, 8, 9 and 11 supporting their presence during heart development. 

**Figure 5:**
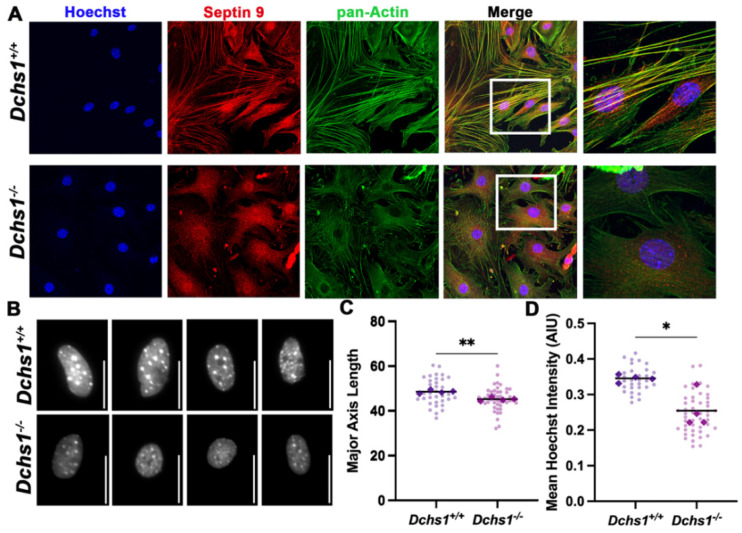
Septin-actin defects in DCHS1-deficient fibroblasts. (**A**) Wildtype (DCHS1^+/+^) and knock-out, (DCHS1^−/−^), cardiac fibroblasts (CFs) were seeded on collagen coated slides and stained for pan-actin (green), septin 9 (red) and nuclei (blue). In DCHS1^−/−^ fibroblasts, actin and SEPT9 no longer co-localize coincident with loss in stress fiber organization and altered cell shape. (**B**) Representative grayscale images of nuclei stained with Hoechst illustrate a rounded phenotype in DCHS-^−/−^, scale bar = 50 pixels. (**C**) Quantification of nuclei major axis length (pixels) and (**D**) Hoechst staining intensity (Arbitrary intensity units, AIU) reveals significant reductions in knockout cells compared to wildtype controls suggesting loss of intracellular tension. 30–50 cells imaged per genotype, N = 4 ** *p* < 0.005, * *p* < 0.05 with Student’s *t*-test.

The well-established function of SEPT9 in actin cytoskeleton dynamics [23] indicated a potential pathway in which changes in DCHS1 at the cell membrane could influence cytoskeletal dynamics through LIX1L-Septin 9 interactions. To initially test the effect of DCHS1 on the septin-actin cytoskeleton, interstitial fibroblasts from DCHS1^−/−^ mice at P0 were plated and stained for Septin 9 (SEPT9) and total actin. As shown in Figure 5, control cells have a near perfect overlay of anisotropic actin stress fibers and SEPT9 filaments. Consistent with these mature stress fibers, we also observe an elongated cell phenotype and stretched nuclei, indicative of intracellular tension. In the DCHS1^−/−^ cells, however, only isotropic actin staining is observed at the cell periphery, with a near-complete loss of filamentous SEPT9 (Figure 5A). Additionally, the cell shape of DCHS1^−/−^ cells resembles that of immature mesenchyme with disorganized actin fibers. Roundness of nuclei, an indicator of loss of intracellular tension [25], was also observed in DCHS1^−/−^ cells, likely as a consequence of cytoskeletal disorganization. Quantification of nuclei length and DNA intensity revealed significant decreases in both in DCHS1-deficient CFs compared to controls (Figure 5B,C). These data support a mechanism by which the DCHS1-LIX1L-SEPT9 (DLS) complex regulates cell shape by promoting actin polymerization and stress fiber formation. 

**Figure 6:**
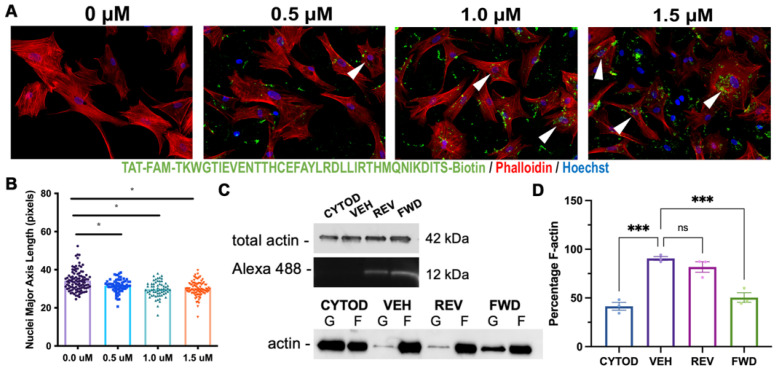
Targeting the LIX1L-SEPT9 binding domain with decoy peptide disrupts F-actin polymerization. (**A**) cVICs treated with increasing doses of SEPT9 decoy peptide conjugated with cell permeant TAT sequence= GRKKRRQRRRPQ, 5-FAM and biotin, diluted in DMSO (0–1.5 μM) for 2 hrs and seeded on collagen coated slides prior to immunocytochemistry staining with phallodin-594 (red) and Hoechst (nuclei = blue). Actin bundling is observed in cells containing peptide (arrowheads). (**B**) Quantification of nuclei major axis length reveals significant reductions in nuclei elongation in response to peptide treatment. N = 3 per treatment group with 20–30 cells per treatment, * *p* < 0.05 with One-Way Anova. (**C**) F/G-actin polymerization assay performed on cVICs seeded at 50% confluence, treated with cytochalasin-D (CYTOD, 1 μM) for 1.5 h and rescued with serum containing media with vehicle (DMSO, VEH), forward (FWD) or reverse (REV) peptide at 1.5 μM. IB of representative total actin and peptide loading and separated F/G-actin content. (**D**) Quantification of F-actin percentage illustrates reduction in F-actin polymerization in FWD peptide-treated cells compared to vehicle. *n* = 3 per treatment, *** *p* < 0.0005 with Ordinary One-Way Anova.

To directly target the LIX1L-SEPT9 interaction in vitro, we synthesized a decoy peptide containing the identified LIX1L binding domain on SEPT9 (TKWGTIEVENTTHCEFAYLRDLLIRTHMQNIKDIT) and fused it to a cell permeating sequence (TAT: GRKKRRQRRRPQ) [26,27], biotin, and 5-carboxyfluorescin (5-FAM) with an excitation maximum in the 488 nm spectral line. This combination allowed for cell permeance and detection of peptide delivery into the cell, which was expected to compete with endogenous SEPT9 to bind LIX1L, resulting in release and destabilization of SEPT9. Dose-dependent decreases in nuclei elongation were observed with increasing peptide dosage, indicating a loss of intracellular actin polymerization and organization (Figure 6A,B). To test whether formation of new F-actin was affected when formation of the DLS complex was disrupted using the inhibitory peptide, cVICs were seeded at 50% confluence and treated with Cytochalasin-D to destabilize actin filaments and then treated with serum containing media with either vehicle (DMSO), forward (FWD) or reverse (REV) peptide to stimulate F-actin polymerization [28]. Actin polymerization was measured with an F/G-actin assay, and significant reductions in F-actin recovery were observed in cells treated with inhibitory peptide compared to reverse peptide or vehicle (Figure 6C,D). These data demonstrate that the LIX1L-SEPT9 interaction is, in part, necessary for the polymerization of F-actin in valve interstitial cells and support a molecular basis for cell organization in the valve tissue.

### 2.4. Consequences of Actin Disorganization in Cells and Tissue

**Figure 7:**
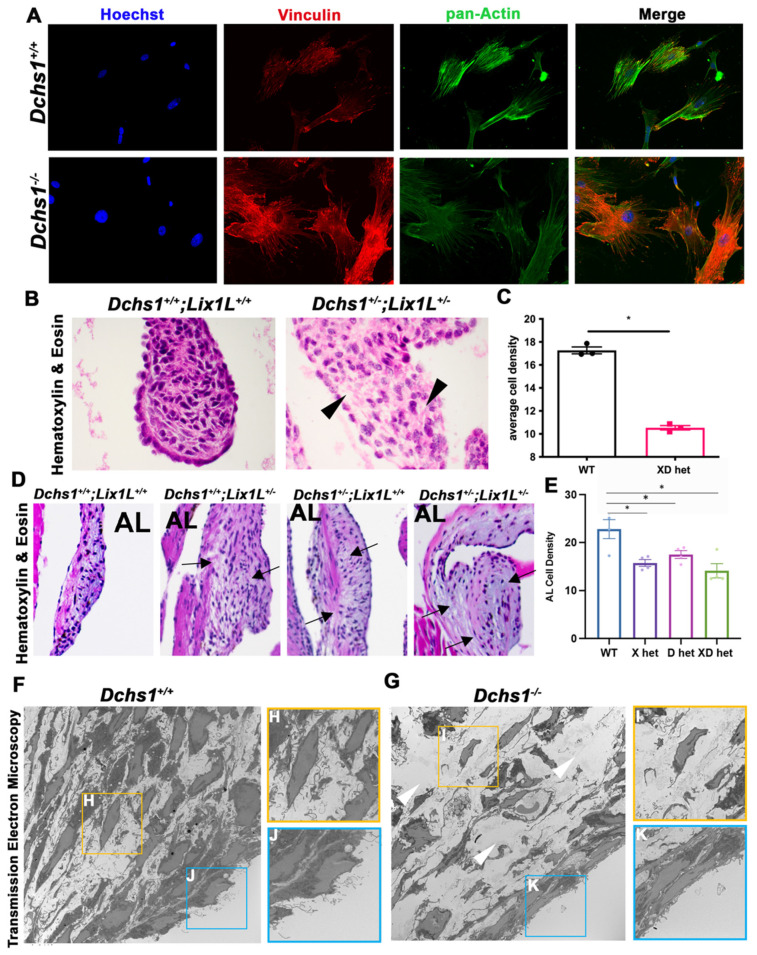
Consequences of actin defects. (**A**) ICC of vinculin (red), pan-actin (green), and nuclei (Hoechst, blue) in wildtype (DCHS1^+/+^) and DCHS1 KO (DCHS1^−/−^) reveals loss of stress fiber formation and vinculin organization and localization to focal adhesions. (**B**) H&E images of wildtype and compound heterozygote P0 valves depict increased density of ECM between interstitial cells (black arrowheads), also observed in (**D**) 11-month-old leaflets of single and compound heterozygote anterior leaflets (arrows). (**C**,**E**) Quantification of cell density measured by cells divided by total leaflet area reveals significant decreases in compound heterozygotes (XD het) compared to controls at P0 and both single and compound heterozygotes in adults. Graphs depict average cell density per animal with error bars of SEM, *n* = 3–4 animals per genotype, * *p* < 0.005. (**F**,**G**) Transmission electron microscopy (TEM) images of wildtype and DCHS1 KO anterior leaflets at P0 show increased ECM content in DCHS1 KOs and less dense interstitium with round nuclei and loss of filopodia-structures (yellow boxes, (**H**,**I**)). Filopodia-like structures appear to be lost along the DCHS1 KO endothelium (blue boxes, **J**,**K**).

Since septin-actin networks have been shown to localize to and promote focal adhesion formation and mechanosensation [29], and focal adhesions connect valve cells to the ECM, we probed for the actin-integrin connecting protein vinculin in primary valve interstitial cells. Figure 7A shows drastic changes in vinculin organization in DCHS1 deficient CFs compared to controls. Consistent with these changes in vinculin, we noticed a correlative reduction in cell density in single (DCHS1^+/−^) or compound heterozygotes (DCHS1^+/−^, LIX1L^+/−^) at 11-months of age. This data suggested a change in ECM deposition or an inability for cells to organize the extracellular matrix as a result of DCHS1/ LIX1L deficiency (Figure 7B–E). We also observed this decrease in cell density in transmission electron microscopy (TEM) imaging of P0 tissue (Figure 7F,G). TEM images depicted less abundant nuclei in DCHS1 KO valves with rounded shape and failure to align in relation to the leaflet’s tension axis. TEM data also revealed that control valve interstitial and endothelial cells contain robust amounts of thin cellular protrusions, which are lost in the DCHS1^−/−^ mice. 

## 3. Discussion

Mitral valve prolapse can lead to serious complications and poses a significant health burden. While there have been many strides towards defining the molecular etiology of MVP, therapeutics beyond surgical intervention have yet to emerge. Thus, efforts to define the genetic and molecular underpinnings are essential to advance effective treatments for patients with MVP. 

It is increasingly recognized that critical stages of valve tissue morphogenesis contribute to mitral valve disease inception. Of such is the point at which the valve leaflets experience increased hemodynamic forces of the growing embryo and tension as the leaflets connect to the papillary when significant thinning and elongation of the valve leaflets becomes evident. Occurring around E17.5 in mice, we suggest that this process leads to proper stratification of ECM as observed in leaflets from healthy humans and wildtype mice [30]. This hypothesis is supported by the current study and various mouse models that exhibit leaflet enlargements as early as postnatal day zero [3,31,32,33,34,35]. In diseased valves, cells are unable to properly stratify the ECM because of genetic and molecular perturbations that engender defective mechano-responses to the imposed tension. It is equally interesting to note that in our models, the posterior leaflet, during development and early neonatal time points, does not show a statistically significant morphological defect, whereas the anterior leaflet does. It is logical to propose that this difference stems from either the timing of morphological events within the valves or the cellular origin of each leaflet. The posterior leaflet begins development at least three days later than the anterior leaflet in mice. Additionally, the anterior leaflet is comprised primarily of endothelial-derived cells. The posterior leaflet, although initially built by endothelial progenitors, matures through the addition of epicardial-derived cells. Thus, the requirement for the DLS complex may not yet be relevant at early neonatal stages in posterior leaflet development and/or may have a different role in the epicardial-derived cell populations. Regardless, the posterior leaflet is profoundly affected in the adult (Figure 4), thus paving the way for additional studies on the role of timing and molecular differences between the anterior and posterior leaflets.

It is compelling that a cytoskeletal mechanism is responsible for proper mechanosensing of valve cells, cell and cell-ECM organization and normal valvulogenesis. Identification of several causal genes in multiple families of MVP patients supports this; these genes include primary cilia genes such as DZIP1 [31], actin binding protein, FLNA [35], focal adhesion interactor, TNS1 [36] and atypical cadherin and DCHS1 [3], among others. Perturbations of each of these genes have significant effects on cell function and confer the cell’s ability to sense, respond to and organize the microenvironment of the valve interstitium. 

This study focused on the MVP causal gene, DCHS1, and defined a novel mechanism that links the atypical cadherin, DCHS1 to LIX1L, SEPT9 and the actin cytoskeleton. Interactions and stability of the DLS protein complex were identified and supported with biochemical assays in vivo and in vitro. A series of yeast two-hybrid screens allowed us to refine each interaction domain and were confirmed by co-IPs and co-expression studies that illustrated the presence of DCHS1-LIX1L-SEPT9 in the developing heart and valve. In addition, mouse epistasis analyses demonstrated a genetic interaction between DCHS1 and LIX1L as compound heterozygosity contributed to a synergistic effect on valve enlargement as early as P0 and in adults. Additionally, compound heterozygotes exhibited significant progression of mitral valve prolapse phenotypes, including myxomatous degeneration, prolapsing leaflets above the annulus and increased left atrial diameter compared to single heterozygotes and control littermates. DCHS1 and/or LIX1L deficiency also led to decreases in septin-9 protein expression in vivo and its disorganization in vitro and motivated the pursual of SEPT9 mediated pathways. 

The septin family and septin-9, in particular, present a multitude of potential pathways, given their ability to interact with and regulate themselves and members of the cytoskeleton [37]. An actin mechanism was pursued following initial immunocytochemical stains of wildtype and DCHS1 deficient cardiac fibroblasts which exhibited significant septin-actin co-expression (Figure 5) compared to scant regionalization to microtubules (data not shown). Furthermore, perturbation of SEPT9 through DCHS1 deficiency or treatment with LIX1L-SEPT9 decoy peptides led to significant reductions in actin organization in vitro. Consequentially, loss of intracellular tension was observed by nuclei roundness and decreased DNA intensity in DCHS1-deficient cells. Although observing the organization of the actin cytoskeleton in highly compacted valve interstitial cells in vivo presents technical challenges, downstream effects on cell structure and tissue organization were identified and should be pursued in future studies. 

The necessity of actin dynamics in shaping the valve early in development has been proposed previously [38,39,40]. Gould et al. showed that mechanical loading confers phenotypic changes as a result of Rho or Rac activation [41] and promotes focal adhesions, stress fiber formation and ECM compaction in valve cell cultures. In vivo, evidence of diseased mitral valves have been observed in *Tensin*-1 [36], *Filamin*-*A* [35] and *Desert hedgehog*-deficient mice [32] and affect focal adhesion formation, actin bundling, and smooth muscle actin expression, respectively. Each of these mice exhibit ECM remodeling defects conferring myxomatous valves, lending credence to actin-related disease mechanisms. 

Our previous studies through TEM imaging demonstrated that interstitial cells align along valve tension points in neonatal and adult valve leaflets [42]. Additionally, in an in vitro system that recapitulates the tension points from the annulus to the papillary, valve cells were able to independently align themselves and deposit and align collagen along the same axis. Thus, there is an illustrated alignment of valve cells that depends on sensed tension. Although it is still possible that cell polarity mechanisms are critical to establish valve cell alignment, it is intriguing to consider whether cell–cell adhesions and downstream effectors are involved and necessary. It is also possible that cell–cell adhesions and cell polarity/division are interdependent; Gloerich et al. showed that E-cadherin aids in mitotic spindle orientation, thus coupling cell division to cell adhesion pathways [43]. In our studies, significant misalignment of valve cells was previously observed in vivo in DCHS1-deficient P0 valves [3] and in TEM images presented here in Figure 7, which displayed rounded and less-aligned nuclei in DCHS1 KO P0 valves in relation to the endothelium. Furthermore, in DCHS1-deficient cells and in cells that are depleted of actin, nuclei roundness and compaction defects were observed. Thus, DCHS1 may be necessary for cell alignment and elongation by promoting septin-actin organization. 

Defects in cell alignment and elongation likely affect concerted ECM organization through focal adhesions. Focal adhesions (FAs) are integrin-based adhesion structures that are associated with the actin cytoskeleton through various proteins. FAs potentiate cell–ECM interactions and, thus, a cell’s sensitivity to biochemical and mechano-environments [44]. FAs, therefore, are crucial structures involved in valve development as cells sense growth factors, ECM substrates and biomechanical forces of tension and hemodynamics to orchestrate tissue growth and shape [45]. Here we probed for vinculin, an actin-binding and integrin-regulating protein that promotes focal adhesion formation and localizes to cell–cell and cell-matrix adhesions. Significant disorganization of vinculin was observed in DCHS1 knockout cells compared to controls. This finding suggests potential downstream focal adhesion defects and altered mechano-sensing, given that vinculin recruitment to cell–cell adhesions is dependent on intercellular forces [46]. Additional support for DLS involvement in FAs is that septins have also been shown to interact with and regulate focal adhesions. Specifically, septin-9 has been shown to regulate Rhoa/FAK signaling [47] and to localize to FA tips that secrete MMP3 and 13 [48]. Though correlative, each of these molecules have been implicated in valve disease and warrant future studies to investigate DLS regulation. 

Lastly, actin dynamics contribute to cell–cell interactions by stabilizing adhesions and promoting cell structures like filopodia. For example, filopodia expressing E-cadherin are essential for mouse embryo compaction and cell shape changes through myosin 10 and f-actin [49]. In the lymphatic valve, co-localization of F-actin and DCHS1 was observed during development and at the tip of filopodia [50] and shown to affect migration and polarization. TEM images of P0 DCHS1-deficient valves revealed defects in what may be actin-containing microvilli or cytonemes, a type of filopodia that are 0.1 µm to 0.5 µm in diameter and can extend up to 150 µm from the cell’s plasma membrane [51]. Cytonemes serve as an expansion of a cell’s surface area and confer unique signaling. By preserving structures through glutaraldehyde fixation [52], cytoneme-specific signaling has been observed involving hedgehog dependent on myosin 10 (HH) [53] and WNT regulated by VANGL2 [54]. Both of these findings are interesting, given our recent investigations of valve phenotypes in *Dhh*- and *Cby1*-deficient mice, which exhibit disruptions in actin and beta-catenin, respectively [32,34]. Additionally, FAT4 and DCHS1 were shown to be targets of GLI zinc finger 2 (GLI2) downstream of HH signaling to regulate villi formation [55] and inhibition of the HH receptor Smoothened (SMO) suppressed increased ECM deposition of cells derived from patients with DCHS1 SNV p.R2330C [21]. Together, these data suggest that cytonemes could foster a unique signaling environment for HH, DCHS1 and other major regulators of valve tissue development.

It is important to briefly note that septins are also able to interact with and regulate the microtubule cytoskeleton. As MVP has been defined as a ciliopathy, a microtubule mechanism is worthy of investigation. In particular, DCHS1 has been observed at ciliary basal bodies along the airway epithelia [56], and significant decreases in cilia lengths were observed in DCHS1 heterozygote mice at P0 [31]. Further evidence for microtubule involvement was observed in early DCHS1-deficient zebrafish embryos affecting microtubule bundling [57]. Additionally, DCHS1 was shown to interact with Ttc28 and Aurora B to control early embryonic cleavages [58] and, interestingly, the binding region was included in our DCHS1-LIX1L interacting domain. In the context of the DCHS1-LIX1L-SEPT9 protein complex presented here, LIX1L has not yet been shown to interact with or regulate microtubules. However, septins 2, 7 and 9 have been observed along ciliary axonemes and in regulation of ciliogenesis [59]. Future studies investigating whether DCHS1-LIX1L-SEPT9 converge on microtubules could prove an additional axis for cilia regulation in the valve. 

**Figure 8:**
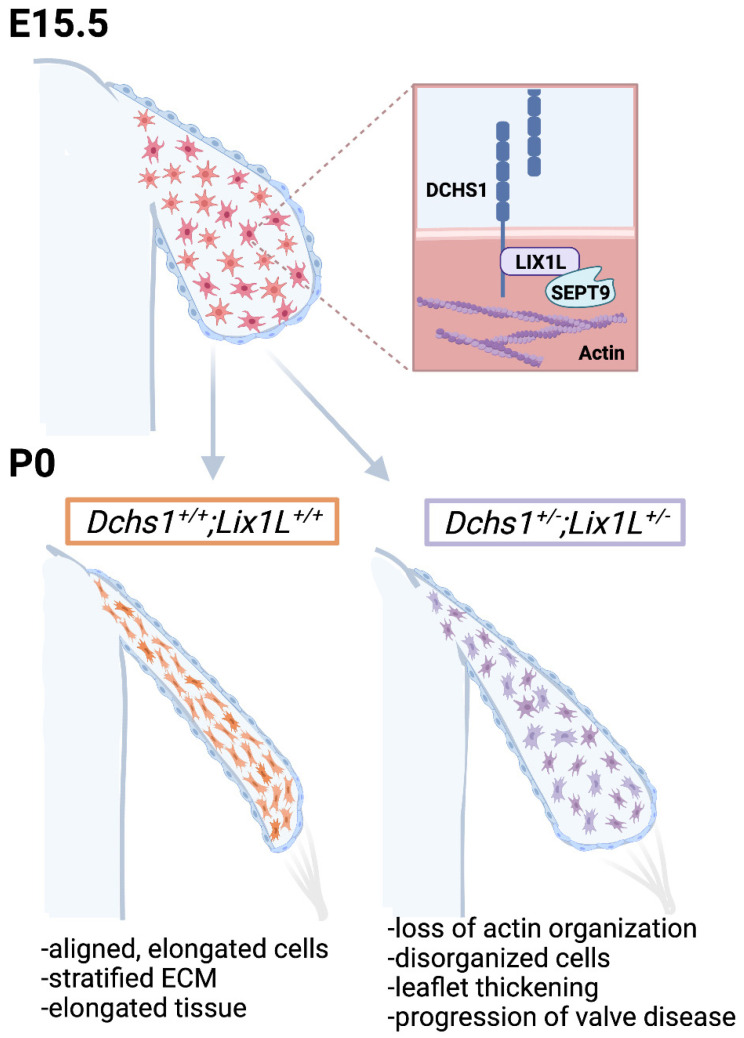
Working model of valve morphogenesis. DCHS1, LIX1L and Sept9 are expressed in undifferentiated mesenchymal cells during embryonic development that are randomly distributed throughout the tissue. As these cells proliferate and increase in density, they encounter neighboring cells and engage cadherin receptors, much like train cars hooking together on a railroad track (ECM). DCHS1-LIX1L-SEPT9 (DLS) complex promotes filamentous actin formation and cell elongation. All cargo attached to the cell membrane, such as integrins and the ECM, then passively assume a similar organization and shape as the cells. This results in aligned, elongated cells and tissues with a stratified ECM. Defects in any step of this process would be anticipated as resulting in disrupted actin organization, disorganized cells, leaflet thickening and progression to a valve pathology.

ECM remodeling in the valve has been regarded as a landmark event critical for proper valvular morphogenesis, which has been reported to be dependent on VIC contractility through alpha-smooth muscle actin activation. However, this may not necessarily be the case based on our results, and we propose a new hypothesis. During embryonic valve development, interstitial cells undergo rapid proliferation. Once they reach a defined density, proliferation slows due to cadherin-driven contact inhibition, and valve thinning begins. We propose that contact inhibition occurs through engagement of DCHS1 or other cadherin proteins on opposing cells. This action, reminiscent of train cars hooking together on a railroad track (ECM), results in activation of the lix1-septin complex, filamentous actin organization and cell elongation. As the cells elongate and organize within the tissue, so do their integrin-interacting ECM cargo attached to the cell membrane. In this way, the ECM becomes organized through a cell-cell interaction that drives actin polymerization (Figure 8). The event of cellular alignment appears to predate maturation of collagen or tissue architecture and brings into question whether cell density and cell–cell contact are key drivers of not only cellular organization but also cellular differentiation into collagen-secreting fibroblastic-like cells. Thus, in the absence of proper intercellular communication, differentiation of interstitial cells does not occur, resulting in retention of embryonic-like proliferative mesenchyme throughout life. If true, as supported by our data, a myxomatous phenotype is not a degenerative disease in patients with MVP as currently thought. Rather, MVP is a defect of cellular differentiation during development, and the adult phenotype results from continued growth of embryonic tissue. 

As our studies focus on developmental defects that give rise to MVP, we are unable to define the precise mechanism by which excess and disorganized ECM occurs at postnatal stages. A common and emergent theme is that developmental defects caused by genetic changes result in structural and morphological alterations in the valve that trigger secondary pathways. These downstream pathways, such as inflammation, likely have a negative impact on the fine balance on synthesis, homeostasis and destruction of ECM in the valve over time. If true, identification of these secondary defects will likely reveal nodes of therapeutic intervention that could be harnessed for patient benefit.

## 4. Methods

### 4.1. Yeast Two-Hybrid Screening Assays

Yeast two-hybrid screening was performed by Hybrigenics Services, S.A.S., Evry, France (http://www.hybrigenics-services.com, 14 Feburary 2022). The coding sequence of fragments containing aa 2962–3191 and 2962–3130 of human DCHS1 (NM_003737.3) were PCR-amplified and cloned into pB27 as a C-terminal fusion to LexA (LexA-bait). The 2962–3191 construct was used as a bait to screen a random-primed human placenta cDNA library, and construct 2962–3130 was used as bait to screen a random-primed human ventricle and embryo heart cDNA library. The libraries are constructed into pP6. The coding sequences for full-length human LIX1L (NM_153713.2) and SEPTIN9 (NM_001113491.2) were PCR-amplified, cloned into pB27 and used as baits to screen the same human ventricle and embryo heart cDNA library. pB27 and pP6 derive from the original pBTM116 and pGADGH plasmids, respectively. For the DCHS1 fragment aa 2962–3191, 110 million clones (11-fold the complexity of the library) were screened using a mating approach with YHGX13 (Y187 ade2-101::loxP-kanMX-loxP, matα) and L40ΔGal4 (mata) yeast strains. 20 His+ colonies were selected on a medium lacking tryptophan, leucine and histidine, and supplemented with 50 mM 3-aminotriazole to handle bait autoactivation. For the DCHS1 fragment aa 2962–3130, 128 million clones (18-fold the complexity of the library) were screened using the same mating approach. 359 His+ colonies were selected on a medium lacking tryptophan, leucine and histidine, and supplemented with 0.5 mM 3-aminotriazole. For LIX1L, 100 million clones (14-fold the complexity of the library) were screened and 365 His+ colonies were selected on medium lacking tryptophan, leucine and histidine, and supplemented with 2 mM 3-aminotriazole. For SEPTIN9, 65 million clones (9-fold the complexity of the library) were screened and 16 His+ colonies were selected on medium lacking tryptophan, leucine and histidine.

The prey fragments of the positive clones were amplified by PCR and sequenced at their 5′ and 3′ junctions. The resulting sequences were used to identify the corresponding interacting proteins in the GenBank database (NCBI) using a fully automated procedure. A confidence score (PBS, for Predicted Biological Score) was attributed to each interaction. The PBS is computed to assess the interaction reliability. This score represents the probability of an interaction to be non-specific. It is an e-value, primarily based on the comparison between the number of independent prey fragments found for an interaction and the chance of finding them at random (background noise). The value varies between 0 and 1. Several thresholds have been arbitrarily defined to rank the results in 4 categories from A (the highest confidence rank) to D. The PBS is also adjusted by integrating the PBS of other interactions from the database of >9000 interaction studies at Hybrigenics in which interaction domains of the involved proteins have been found. These reciprocal interactions found in independent screens are technically very reliable and thus tagged as A, B, or C in order of interaction confidence. 

### 4.2. Co-Immunoprecipatations

For the DCHS1-LIX1L co-immunoprecipitation (Co-IP) assay, HEK293T cells were seeded at 5 × 10^5^ cells per 6-well dish and co-transfected on day 2 with human DNA constructs including DCHS1 with V5 tag (Integrated DNA Technologies) and LIX1L (Myc-DDK-tagged) (OriGene, #RC207865) with FuGENE HD Transfection Reagent (Promega, #E2311). Protein was harvested at 48 h post-transfection using ice-cold RIPA with Halt Protease Inhibitor Cocktail (ThermoFisher, #87786). Anti-FLAG M2 Magnetic Beads (Millipore Sigma, #M8823) were washed with 1 X Tris Buffered Saline (TBS) prior to overnight incubation at 4 °C with 250 ug protein containing DCHS1-V5 and LIX1L-FLAG or DCHS1-V5 alone. Beads were concentrated with a magnetic stand, washed thrice with 1X TBS, and eluted with 2X SDS PAGE Buffer (125 mM Tris pH7, 10% glycerol, 2% SDS, 5% β-mercaptoethanol, and 0.05% bromophenol blue) for 10 min at 98 °C. For LIX1L-SEPT9 co-immunoprecipitations, whole cell lysates harvested from cVICs seeded at 5 × 10^5^ cells per well were treated with peptide conjugated with Biotin and 5-carboxyfluorescein (5-FAM) and containing the SEPT9 binding domain in the forward or reverse orientation diluted in DMSO (FWD:5-FAM-YGRKKRRQRRR-Ahx-TKWGTIEVENTTHCEFAYLRDLLIRTHMQNIKDIT-Lys(Biotin), REV: (5-FAM)YGRKKRRQRRR-Ahx-TIDKINQMHTRILLDRLYAFECHTTNEVEITGWKT-Lys(Biotin)-) (synthesized by GenScript). Protein lysate was incubated with peptide overnight with shaking at 4 °C in 500 uL total volume in 1XTBS-0.1% Tween. Co-IP was performed using 50 uL of Pierce Streptavidin Magnetic Beads (ThermoFisher, #88816) per sample with 1X-TBST as wash buffer and 2X SDS-PAGE Buffer for elution. All immunoblotting (IB) was performed as previously described [33] using primary antibodies: anti-V5 (R&D Systems, #MAB8926), anti-FLAG (Sigma-Aldrich, #F3165, and anti-LIX1L (ProSci, #55-529) diluted 1:1000 in 5% Blotting-Grade Blocker (Bio-Rad, #1706404). 

### 4.3. Echocardiography

Echocardiographic images were acquired in the parasternal long-axis, short-axis, and apical views (40 MHz probe, Visualsonics 3100, Fujifilm, Toronto, ON, Canada). Two-dimensional images were used for quantification of the diameter of the left atrium. Pulse wave mode in conjunction with timing of the cardiac cycle (electrocardiogram) were used to visualize flow patterns at the mitral valve. A total of 3–4 mice were imaged per genotype. Echocardiographic still images and movies are available upon request from the corresponding author.

### 4.4. Valve Morphometrics

For each animal, all sections containing valve tissue were stained with hematoxylin and eosin (H&E), imaged with an Olympus BX40, aligned in ImageJ, and traced to create three-dimensional surface reconstructions in Imaris 9.3 software. Volume measurements of each leaflet were obtained from Imaris for each surface and normalized to total sections, *n* = 4 per genotype. Two dimensional measurements were obtained by measuring length from annulus to tip and width at the base, mid and tip of the anterior and posterior leaflets in ImageJ on 4 sections of 4 animals per genotype. 

### 4.5. In Situ Hybridization

RNA in situ hybridization (ISH) images for *Septins* 2,5,7,8,9, and 11 at E14.5 were obtained from GenePaint (https://gp3.mpg.de/, accessed on 14 February 2022) (Visel, 2004 #5). The set ID and accession# for each generated probe are as follows: *Septin* 2 (EB100, NM_010891), *Septin* 5 (MH585, AF033350), Septin 7 (EH4070, NM_009859), *Septin* 8 (EH1781, NM_033144), *Septin* 9 (EB2571, NM_017380), and *Septin* 11 (EH2064, NM_001009818). All probes were used at E14.5 and depict positive staining in various regions of the heart. 

### 4.6. Cell Culture & Treatments

HEK293T cells (ATCC) were maintained in DMEM with 10% fetal bovine serum and 1% Penicillin-Streptomycin (Millipore Sigma, #11074440001). Chicken valvular interstitial cells (cVICs) were isolated from anterior leaflet of chicken embryos at HH40, as described previously, and maintained in Medium 199 (M199, Invitrogen) containing 5% chicken serum (bioWORLD, #30611183-1), 0.1% insulin transferrin selenium (ITS) (Gibco, 41 400-045) and 1% pen-strep. DCHS1^−/−^ or wildtype cardiac fibroblasts (CFs) were isolated from P0 mouse hearts. In brief, hearts were placed into and minced in ice cold Krebs-Hensleit Buffer with NaHCO_3_ (Sigma Aldrich, K3753) prior to digestion in buffer containing: 0.25% Trypsin (Corning, 25-050-CL), Liberase TM (Roche, 5401020001) and 1M HEPES (Sigma Aldrich, H4034) diluted in Hank’s Balanced Salt Solution (HBSS, Sigma-Aldrich, 55037C). Digested CFs were seeded onto 35 mm dishes with Fibroblast Growth Media (PromoCell, C-23020), washed 24–48 h later, and grown to 85% confluence before passaging. For all experiments, cells were used prior to passage number five and are routinely analyzed for contamination. For F-actin recovery experiments, cVICs were seeded at 3 × 10^5^ cells/six-well dish. On day 1, cells were treated with actin polymerization inhibitor cytochalasin-D (CYTOD) diluted in DMSO at 1 μM for 1.5 h at 37 °C. Cells were stimulated with 4 washes of media containing serum and vehicle (DMSO), forward (FWD) or reverse peptide (REV) (at 1.5 μM in DMSO) prior to harvesting for F/G-actin polymerization assay. HEK293T cells were obtained from ATCC and have been extensively authenticated by ATCC and routinely tested for contamination. 

### 4.7. Immunofluorescence Staining and Imaging

Immunohistochemical staining (IHC) was performed on 5 μm sections of paraformaldehyde fixed, paraffin embedded tissue obtained from E15.5, P0, 4 and 11-month-old wildtype, DCHS1^+/−^, LIX1L^+/−^, or DCHS1^+/−^;LIX1L^+/−^ mice as described previously [32,60,61] using Citrate Based Antigen Unmasking Solution (Vector Labs, #H-3300) and 1% Bovine Serum Albumin for blocking. Immunocytochemistry (ICC) was performed on cVICs or CFs seeded on collagen I coated slides (Corning, #354557), fixed with 4% paraformaldehyde, permeabilized with 0.1% TritonX-100 for 10 min at room temperature, and blocked with 1% bovine serum albumin (BSA) in 1X phosphate buffered saline (PBS). Primary antibodies and dilutions were used as follows: Alexa Fluor 594 Phalloidin (ThermoFisher, A12381, 1:500), pan-Actin (Millipore Sigma, MAB1501), Collagen 1α1 (a gift from Dr. Stanley Hoffman, 1:250), DCHS1 (Novus Biologicals, NBP2-13901, 1:50), LIX1L (Novus Biologicals, NBP1-92074, 1:100), MF20-c (Developmental Studies Hybridoma Bank, 1:50), PECAM1/CD31 (Dianova, DIA-310, 1:50), SEPT9 (ProteinTech, 10769-1-AP, 1:250), Versican (a gift from Dr. Stanley Hoffman, 1:250), and Vinculin (Abcam, ab129002). Secondary antibodies conjugated with Alexa Fluors (488, 568, or Cy5) were purchased from Invitrogen (Carlsbad, CA, USA) and used at 1:100 dilution in 1% BSA in PBS for a 1 h incubation at room temperature. Nuclei were stained with Hoechst (Life Technologies, #H3569, 1:10,000) and slides were cover-slipped with SlowFade Gold antifade reagent (Invitrogen, # S36936). IHCs were performed on at least 3 animals per genotype unless stated otherwise. Images were acquired with either Zeiss Axioscope (Zeiss, Inc., Oberkochen, Germany) or M2 Leica TCS SP5 AOBS Confocal Microscope (Leica, Inc. Wetzlar, Germany) equipped with LAS AF v2.6.3 Build 8173 Acquisition and Analysis Software.

### 4.8. Quantitative Image Analyses

To quantify extracellular matrix intensity, immunofluorescence images were captured at the same exposure time per channel across each animal and cropped in ImageJ to isolate each leaflet and to obtain area measurements. Leaflet images were analyzed with CellProfiler to measure total intensity of collagen 1α1 and versican per leaflet, which was normalized to leaflet area and compared in GraphPad Prism 9. Similarly, cell density was quantified by cropping H&E images of each leaflet to obtain area measurements prior to counting total nuclei per leaflet in CellProfiler and cell density was calculated by total cells divided by area. 

### 4.9. Mouse Husbandry and Analyses

Generation and genotyping of DCHS1 mice was previously described [3,10]. LIX1L heterozygote mice were obtained from EuCOMM (C57BL/6N-A^tm1Brd^ LIX1L^tm1a(EUCOMM)Wtsi^/WtsiBiat, EMMA ID:06287). Genotyping was performed by Transnetyx, Inc. Animals were housed in a 12 h light-dark cycle with water and food ad libitum. All animal experiments were performed under protocols approved by the Institutional Animal Care and Use Committees at the Medical University of South Carolina (protocol #2020-00956). Before cardiac resection, mice were euthanized by decapitation (embryonic stages) or isoflurane (Piramal) induction, followed by cervical dislocation in accordance with the Guide for the Care and Use of Laboratory Animals (NIH publication no. 85–23, revised 1996). Comparisons of the data generated for both male and female sexes showed no appreciable differences. As such, combined data for both sexes are shown. All animal experiments were performed in accordance with IACUC procedures and approved protocol number IACUC-2020-00956.

### 4.10. Actin Polymerization Assay

G-actin/F-actin In Vivo Assay Biochem Kit (Cytoskeleton, Inc., BK037) was used to quantify actin polymerization in cVICs seeded at 3.5 × 105 cells / 6-well dish. Briefly, cells were harvested by scraping with 200 μL of Lysis and F-actin Stabilization Buffer with ATP and protease inhibitor cocktail (LAS2) and lysed for 10 min at 37 °C. Protein lysates were centrifuged at 100,000× *g* at 37°C for 1 h and pellets were dissolved in F-actin depolymerization buffer on ice for 1 h with mixing at 10 min intervals. G- and F-actin samples were denatured with 5X SDS sample buffer and analyzed with western blotting, as previously described. 

### 4.11. Transmission Electron Microscopy (TEM)

Single anterior leaflets from DCHS1^+/+^ (N = 3) or DCHS1^−/−^ (N = 3) mice at P0 were dissected out, fixed in 2% glutaraldehyde and processed for transmission electron microscopy. Samples were rinsed and post-fixed in 1% osmium tetroxide in 0.1 M cocadylate buffer, and thereafter dehydrated in graded acetones and embedded in Epon (Electron Microscopic Sciences, Hatfield, PA, USA). Ultrathin sections were stained with 3% uranyl acetate and 0.2% lead citrate and were examined under a JEOL electron microscope (JEOL, Tokyo, Japan). 

## Data Availability

All datasets will be made publicly available at the time of publication and are accessible at request of the corresponding author.

## Supplementary Materials

The following supporting information can be downloaded at: https://www.mdpi.com/article/10.3390/jcdd9020062/s1. Figure S1: Septin 9 expression is decresed in compound heterozygotes. (**A**) IHC of SEPT9 (red) and nuclei (Hoechst, blue) depicts loss of Septin-9 expression in the interstitium of compound hets (DCHS1^+/−^; LIX1L^+/−^) compared to controls (DCHS^+/+^; LIX1L^+/+^). (**B**) Quantification of staining intensity reveals significant decreases in SEPT9 expression. *n* = 3 per genotype, ** = *p* < 0.005 with Student’s *t*-test; Figure S2: Compound heterozygote mice display mitral valve prolapse and increased left atrium diameter throughout adulthood. Quantification of left atrium (LA) diameter measured in 2D images in M-Mode exhibit significant increases in compound heterozygotes compared to wildtype controls at 3, 6, and 11 months of age. N = 3, 4 animals per genotype, * = *p* < 0.05, ** = *p* < 0.005 with a One-Way Anova, graphs depict individual data points, mean and SEM.

## References

[1] Gammie J.S., Chikwe J., Badhwar V., Thibault D.P., Vemulapalli S., Thourani V.H., Gillinov M., Adams D.H., Rankin J.S., Ghoreishi M. (2018). Isolated Mitral Valve Surgery: The Society of Thoracic Surgeons Adult Cardiac Surgery Database Analysis. Ann. Thorac. Surg..

[2] Grigioni F., Tribouilloy C., Avierinos J.F., Barbieri A., Ferlito M., Trojette F., Tafanelli L., Branzi A., Szymanski C., Habib G. (2008). Outcomes in Mitral Regurgitation Due to Flail Leaflets: A Multicenter European Study. JACC Cardiovasc. Imaging.

[3] Durst R., Sauls K., Peal D.S., DeVlaming A., Toomer K., Leyne M., Salani M., Talkowski M., Brand H., Perrocheau M. (2015). Mutations in DCHS1 cause mitral valve prolapse. Nature.

[4] Clemenceau A., Bérubé J.-C., Bélanger P., Gaudreault N., Lamontagne M., Toubal O., Clavel M.-A., Capoulade R., Mathieu P., Pibarot P. (2017). Deleterious variants in DCHS1 are prevalent in sporadic cases of mitral valve prolapse. Mol. Genet. Genom. Med..

[5] Rogulja D., Rauskolb C., Irvine K.D. (2008). Morphogen Control of Wing Growth through the Fat Signaling Pathway. Dev. Cell.

[6] Han M.-R., Han K.-M., Kim A., Kang W., Kang Y., Kang J., Won E., Tae W.-S., Cho Y., Ham B.-J. (2019). Whole-exome sequencing identifies variants associated with structural MRI markers in patients with bipolar disorders. J. Affect. Disord..

[7] Cellini E., Vetro A., Conti V., Marini C., Doccini V., Clementella C., Parrini E., Giglio S., Della Monica M., Fichera M. (2019). Multiple genomic copy number variants associated with periventricular nodular heterotopia indicate extreme genetic heterogeneity. Eur. J. Hum. Genet..

[8] Klaus J., Kanton S., Kyrousi C., Ayo-Martin A.C., Di Giaimo R., Riesenberg S., O’Neill A.C., Camp J.G., Tocco C., Santel M. (2019). Altered neuronal migratory trajectories in human cerebral organoids derived from individuals with neuronal heterotopia. Nat. Med..

[9] Lodge E.J., Xekouki P., Silva T.S., Kochi C., Longui C.A., Faucz F.R., Santambrogio A., Mills J.L., Pankratz N., Lane J. (2020). Requirement of FAT and DCHS protocadherins during hypothalamic-pituitary development. JCI Insight.

[10] Mao Y., Mulvaney J., Zakaria S., Yu T., Morgan K.M., Allen S., Basson M.A., Francis-West P., Irvine K.D. (2011). Characterization of a DCHS1 mutant mouse reveals requirements for DCHS1-Fat4 signaling during mammalian development. Development.

[11] Crespo-Enriquez I., Hodgson T., Zakaria S., Cadoni E., Shah M., Allen S., Al-Khishali A., Mao Y., Yiu A., Petzold J. (2019). DCHS1-Fat4 regulation of osteogenic differentiation in mouse. Development.

[12] Mao Y., Kuta A., Crespo-Enriquez I., Whiting D., Martin T., Mulvaney J., Irvine K.D., Francis-West P. (2016). DCHS1–Fat4 regulation of polarized cell behaviours during skeletal morphogenesis. Nat. Commun..

[13] Cho E., Irvine K.D. (2004). Action of fat, four-jointed, dachsous and dachs in distal-to-proximal wing signaling. Development.

[14] Mao Y., Rauskolb C., Cho E., Hu W.-L., Hayter H., Minihan G., Katz F.N., Irvine K.D. (2006). Dachs: An unconventional myosin that functions downstream of Fat to regulate growth, affinity and gene expression in Drosophila. Development.

[15] Bosveld F., Bonnet I., Guirao B., Tlili S., Wang Z., Petitalot A., Marchand R., Bardet P.-L., Marcq P., Graner F. (2012). Mechanical Control of Morphogenesis by Fat/Dachsous/Four-Jointed Planar Cell Polarity Pathway. Science.

[16] Li J., Zhu X., Zhang M., Zhang Y., Ye S., Leng Y., Yang T., Kong L., Zhang H. (2021). Limb expression 1-like (LIX1L) protein promotes cholestatic liver injury by regulating bile acid metabolism. J. Hepatol..

[17] Nakamura S., Kahyo T., Tao H., Shibata K., Kurabe N., Yamada H., Shinmura K., Ohnishi K., Sugimura H. (2015). Novel roles for LIX1L in promoting cancer cell proliferation through ROS1-mediated LIX1L phosphorylation. Sci. Rep..

[18] Iliadis C., Baldus S., Kalbacher D., Boekstegers P., Schillinger W., Ouarrak T., Zahn R., Butter C., Zuern C.S., Von Bardeleben R.S. (2020). Impact of left atrial diameter on outcome in patients undergoing edge-to-edge mitral valve repair: Results from the German TRAnscatheter Mitral valve Interventions (TRAMI) registry. Eur. J. Heart Fail..

[19] Cameli M., Incampo E., Mondillo S. (2017). Left atrial deformation: Useful index for early detection of cardiac damage in chronic mitral regurgitation. IJC Heart Vasc..

[20] Aikawa E., Schoen F.J. (2014). Calcific and Degenerative Heart Valve Disease. Cellular and Molecular Pathobiology of Cardiovascular Disease.

[21] Neri T., Hiriart E., van Vliet P., Faure E., Norris R.A., Farhat B., Jagla B., Lefrancois J., Sugi Y., Moore-Morris T. (2019). Human pre-valvular endocardial cells derived from pluripotent stem cells recapitulate cardiac pathophysiological valvulogenesis. Nat. Commun..

[22] Mostowy S., Cossart P. (2012). Septins: The fourth component of the cytoskeleton. Nat. Rev. Mol. Cell Biol..

[23] Dolat L., Hu Q., Spiliotis E.T. (2013). Septin functions in organ system physiology and pathology. Biol. Chem..

[24] Moore K., Moore R., Wang C., Norris R.A. (2020). Tugging at the Heart Strings: The Septin Cytoskeleton in Heart Development and Disease. J. Cardiovasc. Dev. Dis..

[25] Versaevel M., Grevesse T., Gabriele S. (2012). Spatial coordination between cell and nuclear shape within micropatterned endothelial cells. Nat. Commun..

[26] Frankel A.D., Pabo C.O. (1988). Cellular uptake of the tat protein from human immunodeficiency virus. Cell.

[27] Green M., Loewenstein P.M. (1988). Autonomous functional domains of chemically synthesized human immunodeficiency virus tat trans-activator protein. Cell.

[28] Schulze N., Graessl M., Soares A.B., Geyer M., Dehmelt L., Nalbant P. (2014). FHOD1 regulates stress fiber organization by controlling transversal arc and dorsal fiber dynamics. J. Cell Sci..

[29] Dolat L., Hunyara J.L., Bowen J.R., Karasmanis E., Elgawly M., Galkin V.E., Spiliotis E.T. (2014). Septins promote stress fiber–mediated maturation of focal adhesions and renal epithelial motility. J. Cell Biol..

[30] Hinton R.B., Lincoln J., Deutsch G.H., Osinska H., Manning P.B., Benson D.W., Yutzey K.E. (2006). Extracellular Matrix Remodeling and Organization in Developing and Diseased Aortic Valves. Circ. Res..

[31] Toomer K.A., Yu M., Fulmer D., Guo L., Moore K.S., Moore R., Drayton K.D., Glover J., Peterson N., Ramos-Ortiz S. (2019). Primary cilia defects causing mitral valve prolapse. Sci. Transl. Med..

[32] Fulmer D., Toomer K.A., Glover J., Guo L., Moore K., Moore R., Stairley R., Gensemer C., Abrol S., Rumph M.K. (2020). Desert hedgehog-primary cilia cross talk shapes mitral valve tissue by organizing smooth muscle actin. Dev. Biol..

[33] Moore K., Fulmer D., Guo L., Koren N., Glover J., Moore R., Gensemer C., Beck T., Morningstar J., Stairley R. (2021). PDGFRα: Expression and Function during Mitral Valve Morphogenesis. J. Cardiovasc. Dev. Dis..

[34] Guo L., Beck T., Fulmer D., Ramos-Ortiz S., Glover J., Wang C., Moore K., Gensemer C., Morningstar J., Moore R. (2021). DZIP1 regulates mammalian cardiac valve development through a Cby1-beta-catenin mechanism. Dev. Dyn..

[35] Sauls K., de Vlaming A., Harris B.S., Williams K., Wessels A., Levine R.A., Slaugenhaupt S.A., Goodwin R.L., Pavone L.M., Merot J. (2012). Developmental basis for filamin-A-associated myxomatous mitral valve disease. Cardiovasc. Res..

[36] Dina C., Bouatia-Naji N., Tucker N., Delling F.N., Toomer K., Durst R., Perrocheau M., Fernandez-Friera L., Solis J., PROMESA Investigators (2015). Genetic association analyses highlight biological pathways underlying mitral valve prolapse. Nat. Genet..

[37] Spiliotis E.T., Nakos K. (2021). Cellular functions of actin- and microtubule-associated septins. Curr. Biol..

[38] Butcher J.T., McQuinn T.C., Sedmera D., Turner D., Markwald R.R. (2007). Transitions in Early Embryonic Atrioventricular Valvular Function Correspond with Changes in Cushion Biomechanics That Are Predictable by Tissue Composition. Circ. Res..

[39] Butcher J.T., Nerem R.M. (2007). Valvular endothelial cells and the mechanoregulation of valvular pathology. Philos. Trans. R. Soc. B Biol. Sci..

[40] Yalcin H.C., Shekhar A., McQuinn T.C., Butcher J.T. (2011). Hemodynamic patterning of the avian atrioventricular valve. Dev. Dyn..

[41] Gould R.A., Yalcin H.C., MacKay J.L., Sauls K., Norris R., Kumar S., Butcher J.T. (2016). Cyclic Mechanical Loading Is Essential for Rac1-Mediated Elongation and Remodeling of the Embryonic Mitral Valve. Curr. Biol..

[42] De Vlaming A., Sauls K., Hajdu Z., Visconti R.P., Mehesz A.N., Levine R.A., Slaugenhaupt S.A., Hagège A., Chester A.H., Markwald R.R. (2012). Atrioventricular valve development: New perspectives on an old theme. Differentiation.

[43] Gloerich M., Bianchini J.M., Siemers K.A., Cohen D.J., Nelson W.J. (2017). Cell division orientation is coupled to cell–cell adhesion by the E-cadherin/LGN complex. Nat. Commun..

[44] Geiger B., Spatz J.P., Bershadsky A.D. (2009). Environmental sensing through focal adhesions. Nat. Rev. Mol. Cell Biol..

[45] Pagnozzi L.A., Butcher J.T. (2017). Mechanotransduction Mechanisms in Mitral Valve Physiology and Disease Pathogenesis. Front. Cardiovasc. Med..

[46] Le Duc Q., Shi Q., Blonk I., Sonnenberg A., Wang N., Leckband D., de Rooij J. (2010). Vinculin potentiates E-cadherin mechanosensing and is recruited to actin-anchored sites within adherens junctions in a myosin II–dependent manner. J. Cell Biol..

[47] Zeng Y., Cao Y., Liu L., Zhao J., Zhang T., Xiao L., Jia M., Tian Q., Yu H., Chen S. (2019). SEPT9_i1 regulates human breast cancer cell motility through cytoskeletal and RhoA/FAK signaling pathway regulation. Cell Death Dis..

[48] Marcus J., Bejerano-Sagie M., Patterson N., Bagchi S., Verkhusha V., Connolly D., Goldberg G.L., Golden A., Sharma V.P., Condeelis J. (2019). Septin 9 isoforms promote tumorigenesis in mammary epithelial cells by increasing migration and ECM degradation through metalloproteinase secretion at focal adhesions. Oncogene.

[49] Fierro-González J.C., White M.D., Silva J.C., Plachta N. (2013). Cadherin-dependent filopodia control preimplantation embryo compaction. Nat. Cell Biol..

[50] Pujol F., Hodgson T., Martinez-Corral I., Prats A.-C., Devenport D., Takeichi M., Genot E., Mäkinen T., Francis-West P., Garmy-Susini B. (2017). Dachsous1–Fat4 Signaling Controls Endothelial Cell Polarization During Lymphatic Valve Morphogenesis—Brief Report. Arter. Thromb. Vasc. Biol..

[51] Casas-Tintó S., Portela M. (2019). Cytonemes, Their Formation, Regulation, and Roles in Signaling and Communication in Tumorigenesis. Int. J. Mol. Sci..

[52] Bodeen W.J., Marada S., Truong A., Ogden S.K. (2017). A fixation method to preserve cultured cell cytonemes facilitates mechanistic interrogation of morphogen transport. Development.

[53] Hall E.T., Dillard M.E., Stewart D.P., Zhang Y., Wagner B., Levine R.M., Pruett-Miller S.M., Sykes A., Temirov J., Cheney R.E. (2021). Cytoneme delivery of Sonic Hedgehog from ligand-producing cells requires Myosin 10 and a Dispatched-BOC/CDON co-receptor complex. eLife.

[54] Brunt L., Greicius G., Rogers S., Evans B.D., Virshup D.M., Wedgwood K.C.A., Scholpp S. (2021). Vangl2 promotes the formation of long cytonemes to enable distant Wnt/β-catenin signaling. Nat. Commun..

[55] Rao-Bhatia A., Zhu M., Yin W.-C., Coquenlorge S., Zhang X., Woo J., Sun Y., Dean C.H., Liu A., Hui C.-C. (2020). Hedgehog-Activated Fat4 and PCP Pathways Mediate Mesenchymal Cell Clustering and Villus Formation in Gut Development. Dev. Cell.

[56] Dau C., Fliegauf M., Omran H., Schlensog M., Dahl E., van Roeyen C.R., Kriz W., Moeller M.J., Braun G.S. (2016). The atypical cadherin Dachsous1 localizes to the base of the ciliary apparatus in airway epithelia. Biochem. Biophys. Res. Commun..

[57] Li-Villarreal N., Forbes M.M., Loza A.J., Chen J., Ma T., Helde K., Moens C.B., Shin J., Sawada A., Hindes A.E. (2015). Dachsous1b cadherin regulates actin and microtubule cytoskeleton during early zebrafish embryogenesis. Development.

[58] Chen J., Castelvecchi G.D., Li-Villarreal N., Raught B., Krezel A.M., McNeill H., Solnica-Krezel L. (2018). Atypical Cadherin Dachsous1b Interacts with Ttc28 and Aurora B to Control Microtubule Dynamics in Embryonic Cleavages. Dev. Cell.

[59] Ghossoub R., Hu Q., Failler M., Rouyez M.-C., Spitzbarth B., Mostowy S., Wolfrum U., Saunier S., Cossart P., Nelson W.J. (2013). Septins 2, 7, and 9 and MAP4 co-localize along the axoneme in the primary cilium and control ciliary length. J. Cell Sci..

[60] Toomer K.A., Fulmer D., Guo L., Drohan A., Peterson N., Swanson P., Brooks B., Mukherjee R., Body S., Lipschutz J.H. (2017). A role for primary cilia in aortic valve development and disease. Dev. Dyn..

[61] Fulmer D., Toomer K., Guo L., Moore K., Glover J., Moore R., Stairley R., Lobo G., Zuo X., Dang Y. (2019). Defects in the Exocyst-Cilia Machinery Cause Bicuspid Aortic Valve Disease and Aortic Stenosis. Circulation.

