# Supplementary material for: DCHS1, Lix1L, and the Septin Cytoskeleton: Molecular and Developmental Etiology of Mitral Valve Prolapse
Source: J Cardiovasc Dev Dis. 2022 Feb 17;9(2):62. doi: 10.3390/jcdd9020062 (PMC8874669; doi:10.3390/jcdd9020062)
Supplement: Supplementary file 1 [file jcdd-09-00062-s001.zip › jcdd-1557585-supplementary.pdf]

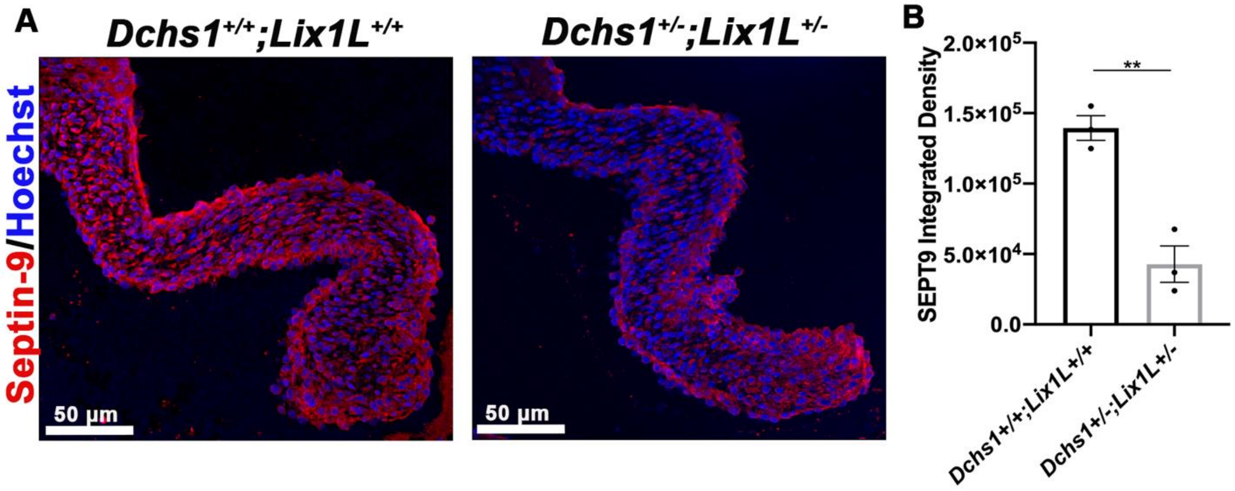

Figure S1. Septin 9 expression is decresed in compound heterozygotes. (A) IHC of SEPT9 (red) and nuclei (Hoechst, blue) depicts loss of Septin-9 expression in the interstitium of compound hets (*Dchs1*$^{+/-}$*;Lix1L*$^{+/-}$) compared to controls (*Dchs1*$^{+/+}$*;Lix1L*$^{+/+}$*).* (**B**) Quantification of staining intensity reveals significant decreases in SEPT9 expression. n=3 per genotype,**=p<0.005 with Student's t-test.

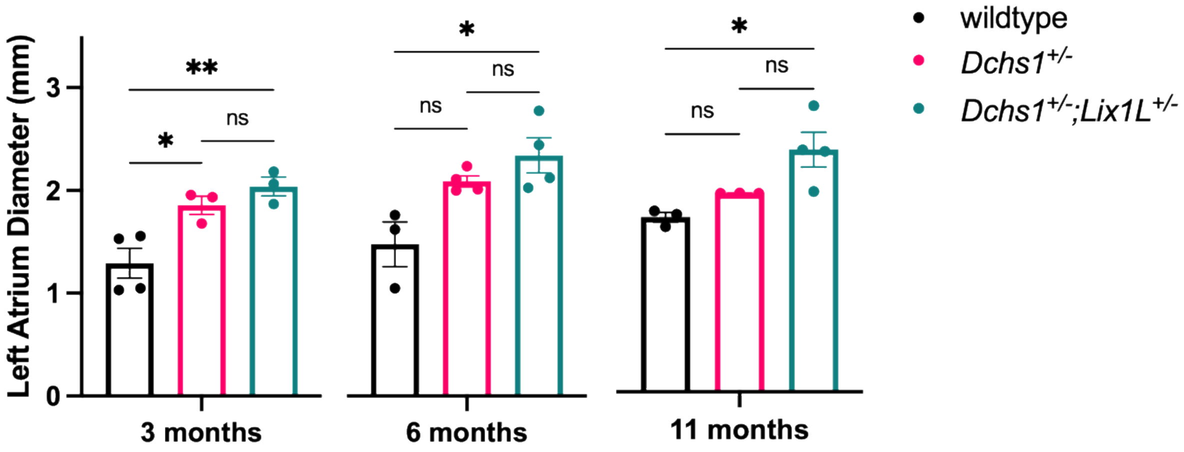

Figure S2. Compound heterozygote mice display mitral valve prolapse and increased left atrium diameter throughout adulthood. Quantification of left atrium (LA) diameter measured in 2D images in M-Mode exhibit significant increases in compound heterozygotes compared to wildtype controls at 3, 6, and 11 months of age. N=3,4 animals per genotype, *=p<0.05, **=p<0.005 with a One-Way Anova, graphs depict individual data points, mean and SEM.

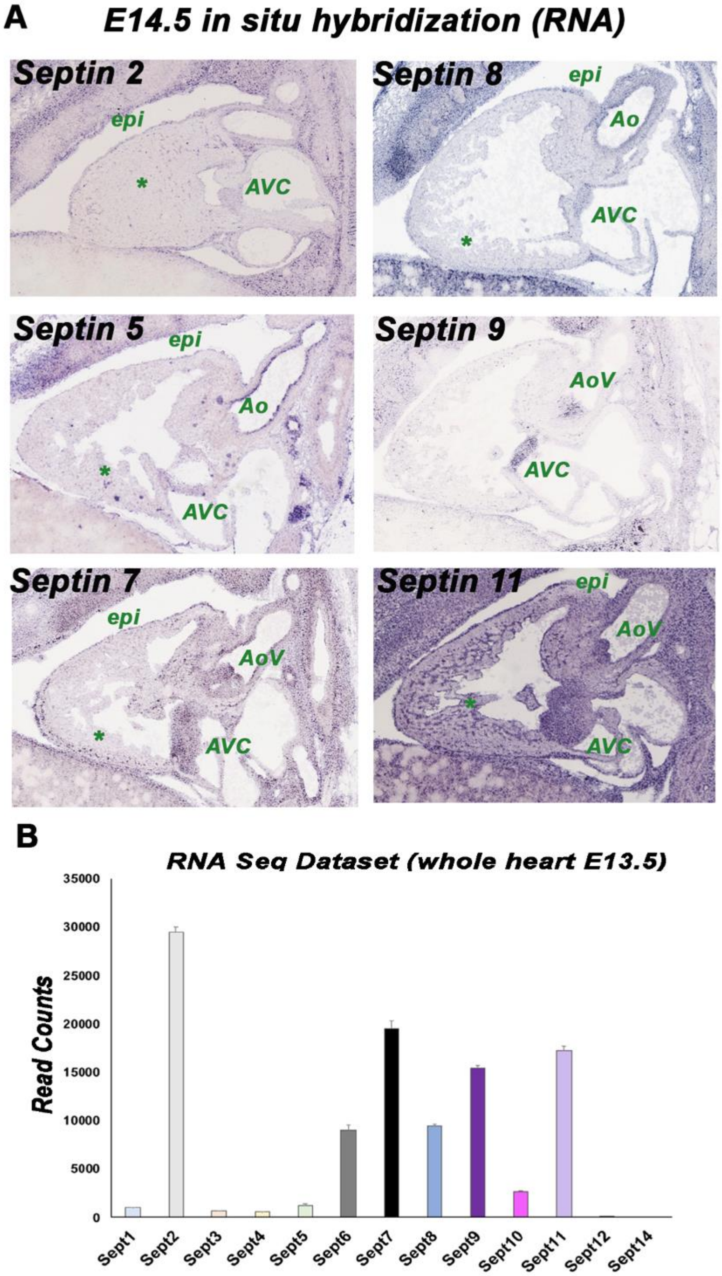

Figure S3. *Septin* RNA expression in developing hearts. (A) In situ hybridization (ISH) data obtained from GenePaint of wildtype E14.5 hearts depict expression of Septin 2, 5, 7, 8, 9, and 11 RNA in various regions (AVC=atrioventricular canal, Ao=aorta, Ao= Aortic Valve, epi=epicardium, *=myocardium). **(B)** RNAseq data of whole heart E13.5 supports expression of Sept 2, 5, 7, 8, 9, 10, and 11.
